# Recovery of layered tissue optical properties from spatial frequency-domain spectroscopy and a deterministic radiative transport solver

**DOI:** 10.1117/1.JBO.24.7.071607

**Published:** 2018-11-19

**Authors:** Sean T. Horan, Adam R. Gardner, Rolf Saager, Anthony J. Durkin, Vasan Venugopalan

**Affiliations:** a University of California, Department of Mathematics, Irvine, California, United States; b University of California, Beckman Laser Institute, Laser Microbeam and Medical Program, Irvine, California, United States; c University of California, Department of Chemical Engineering and Materials Science, Irvine, California, United States; d Linköping University, Department of Biomedical Engineering, Linköping, Sweden; e University of California, Department of Biomedical Engineering, Irvine, California, United States

**Keywords:** inverse problem, spatial frequency domain, staged inversion, analytic solver

## Abstract

We present a method to recover absorption and reduced scattering spectra for each layer of a two-layer turbid media from spatial frequency-domain spectroscopy data. We focus on systems in which the thickness of the top layer is less than the transport mean free path $\left(0.1-0.8l^{*}\right)$. We utilize an analytic forward solver, based upon the N’th-order spherical harmonic expansion with Fourier decomposition $\left({\mathrm{SHEF}}_{N}\right)$ method in conjunction with a multistage inverse solver. We test our method with data obtained using spatial frequency-domain spectroscopy with 32 evenly spaced wavelengths within λ=450 to 1000 nm on six-layered tissue phantoms with distinct optical properties. We demonstrate that this approach can recover absorption and reduced scattering coefficient spectra for both layers with accuracy comparable with current Monte Carlo methods but with lower computational cost and potential flexibility to easily handle variations in parameters such as the scattering phase function or material refractive index. To our knowledge, this approach utilizes the most accurate deterministic forward solver used in such problems and can successfully recover properties from a two-layer media with superficial layer thicknesses.

## 1 Introduction

Spatial frequency domain imaging and spectroscopy (SFDI/SFDS) are optical reflectance-based methods that, when used in combination with quantitative radiative transport models, have provided biomedical optics researchers a powerful means to derive quantitative measures of tissue structure and composition.^1^ Using relatively simple modeling approaches, SFDI has been useful for informing a wide range of diverse biomedical applications ranging from assessment of cerebral hemodynamics in a mouse model of Alzheimers disease^2^ to detection of early modes of failure in tissue transfer flaps^3^ and assessment of burn wound severity.^3^^,^^4^ However, the ability to employ reflectance data acquired at multiple spatial frequencies has been thus far underutilized in terms of enabling optical tomography^5^ and the analysis of layered tissue systems.^6^ The success of early efforts to derive some degree of depth-resolved information has been constrained by the limitations of the standard diffusion approximation (SDA) to the radiative transport equation (RTE), which is typically used for optical property recovery and reconstruction.

Here, we examine the use of SFDS to provide data necessary to determine the optical properties of layered turbid samples having characteristic spatial scales (layer thicknesses) smaller than the transport mean free path $l^{*}$. We apply a high-order RTE approximation that employs a full spherical harmonic functional expansion,^7^ in conjunction with a multistage optimization algorithm,^8^^,^^9^ to estimate layered optical properties from SFDS datasets^10^ acquired from the measurement of layered tissue-simulating phantoms.^11^ The acquisition of SFDS datasets on such phantom systems enables a unique opportunity to explore the impact of illumination wavelength and spatial frequency^12^ on the ability to recover the optical properties of layered systems on spatial scales smaller than $l^{*}$.

Several groups have examined the use of deterministic radiative transport models to quantify optical properties of layered tissues using optical reflectance-based methods including spatially resolved reflectance,^13^ time-resolved reflectance,^14^^,^^15^ temporal frequency-domain reflectance,^16^^,^^17^ and spatial frequency-domain^6^ reflectance. In these studies, the reflectance data is analyzed using the SDA to the RTE. However, the applicability of the SDA is well known to be limited to media whose reduced scattering coefficient dominates that of absorption $\left(\mu_{s}^{\prime }/\mu_{a}\gg 1\right)$ and for spatial scales L larger than the transport mean free path $\left[L≳l^{*}=1/(\mu_{a}+\mu_{s}^{\prime })\right]$. As such, the SDA performs poorly when applied to systems with thin layers, i.e., layer thicknesses $\leq l^{*}$. Specifically for SFDI, Weber and coworkers used the SDA to solve the inverse problem in a two-layered media.^6^ Examination of systems with characteristic layer thicknesses $>2l^{*}$ using an SDA model proved successful in recovering the optical properties ($\mu_{s}^{\prime }$ in particular) at a single wavelength for the top layer of a two-layer system. Interestingly, they found the use of top layer thickness estimates within 25% of the true value still produced useful results.

An alternate approach explored by several groups is to pair noninvasive optical measurements of layered tissues with Monte Carlo (MC)-based radiative transport solvers to determine layered optical properties. In these studies, the computational expense of conventional Monte Carlo simulations was managed using perturbation,^18^ scaled,^19^^,^^20^ or lookup table (LUT)^21^ approaches. Attempts to use MC simulations directly to recover the properties of two-layered media by Seo and coworkers^18^ (using perturbation MC) and Liu and Ramanujam^20^ (using scaled MC) have been met with some success; with error rates in the recovery of top layer optical properties at a single wavelength in the range of 15% to 20% for top layer thicknesses ≳200  μm; $\text{roughly}>l^{*}/5$ in these systems.

Due to their simplicity and speed, LUT-based approaches^21^ have also been explored for determining layered media optical properties. However, given the potential high dimensionality of the parameter space, many assumptions are often adopted to reduce complexity. These methods and others^22^^,^^23^ have focused on the recovery of layered tissue optical properties using contact fiber optic probes with small source–detector separations in conjunction with multilayered optical transport models in systems with layer thicknesses as small as 70  μm. To reduce the high dimensionality of the LUTs used, these groups assume equivalent scattering spectra of the multiple layers or known layer thicknesses.^21^^,^^23^ When considering multispectral data, performance has been mixed in recovering biological characteristics. For instance, optical properties have been used to reliably infer oxygen saturation with a relative error of 5% to 12% in one case.^23^ In other cases, where LUT approaches have provided strong performance, parameter limitations have been introduced, such as the investigation into a small number of wavelengths^21^ or the use of simplifying assumptions, such as equivalent-reduced scattering spectra across layers and known-layer thickness.

Given the limitations of the SDA, as well as the storage and/or computational complexities inherent with MC methods, we sought to develop an approach using a deterministic radiative transport solver suitable for the analysis of SFDS data on spatial scales smaller than the transport mean free path. We have adopted the forward solver developed by Gardner and coworkers,^7^ which provides an approximate solution to the RTE by performing an N’th-order spherical harmonic expansion with Fourier decomposition (${\mathrm{SHEF}}_{N}$) resulting in a system of ${\left(N+1\right)}^{2}$ coupled ordinary differential equations. This formulation, unlike the traditional $P_{N}$ approximation, does not assume azimuthal asymmetry for the angular distribution of the radiance. We have generalized ${\mathrm{SHEF}}_{N}$ for application to layered media and coupled it to a staged inversion algorithm. Data from different spatial frequencies, acquired using an SFDS device,^10^^,^^24^ are used in the different stages to optimize sensitivity and specificity to the layered optical properties of interest.^12^

## 2 Methods and Materials

### 2.1 Spatial Frequency Domain Spectroscopy Instrument and Data Acquisition

**Fig. 1:**
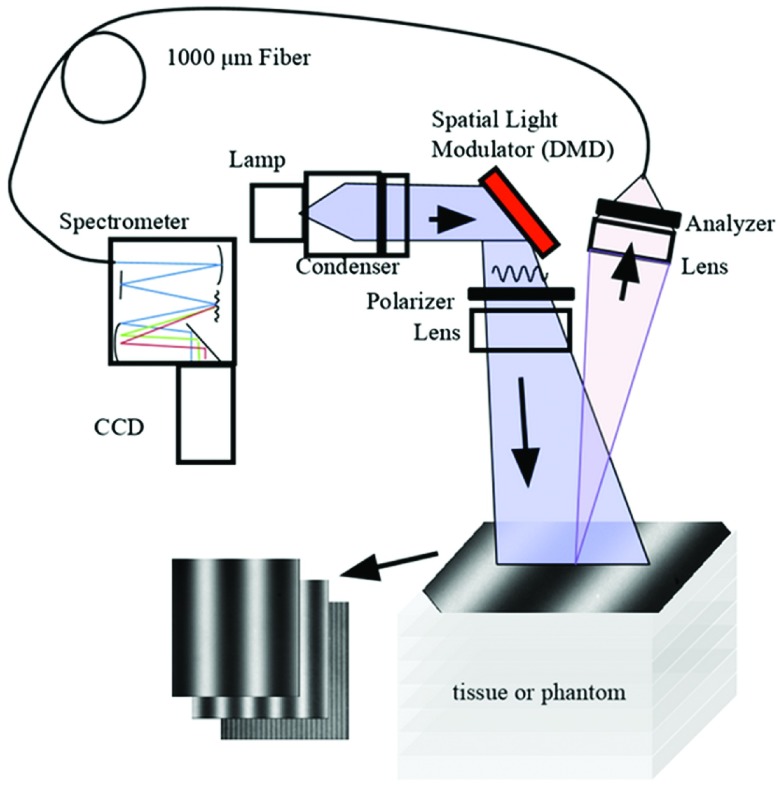
SFDS device and tissue phantom. Broadband optical illumination (λ=450 to 1000 nm) and sinusoidal spatial modulations with frequencies from 0 to $0.5\;{\mathrm{mm}}^{-1}$ using phase shifts of 0, 2π/3, and 4π/3 are projected onto a layered siloxane phantom. The resulting images are captured and the strength of the reflected AC modulation is determined.

Reflectance measurements on a set of layered phantoms spanning a range of optical properties and top layer thicknesses were collected using a SFDS instrument as previously described.^10^ Briefly, multispectral spatial frequency-dependent reflectance data was acquired using a broadband light source that is sinusoidally intensity-modulated using a spatial-light modulator and projected onto the turbid phantoms. Fifty-one evenly spaced spatial frequencies were projected onto each sample, ranging from 0 (uniform, planar illumination) to $0.5\;{\mathrm{mm}}^{-1}$, at $0.01\;{\mathrm{mm}}^{-1}$ intervals. At each spatial frequency, the illumination pattern was projected at three evenly spaced phase shifts: 0, 2π/3, and 4π/3. This approach allows the use of a simple demodulation scheme to determine the AC magnitude of the spatial frequency-dependent reflectance at a single location.^25^ The spectral range of the data collected by this SFDS instrument for this particular investigation spans λ=450 to 1000 nm, at ∼1  nm spectral resolution (Fig. 1).

### 2.2 Layered Tissue Phantoms

**Fig. 2:**
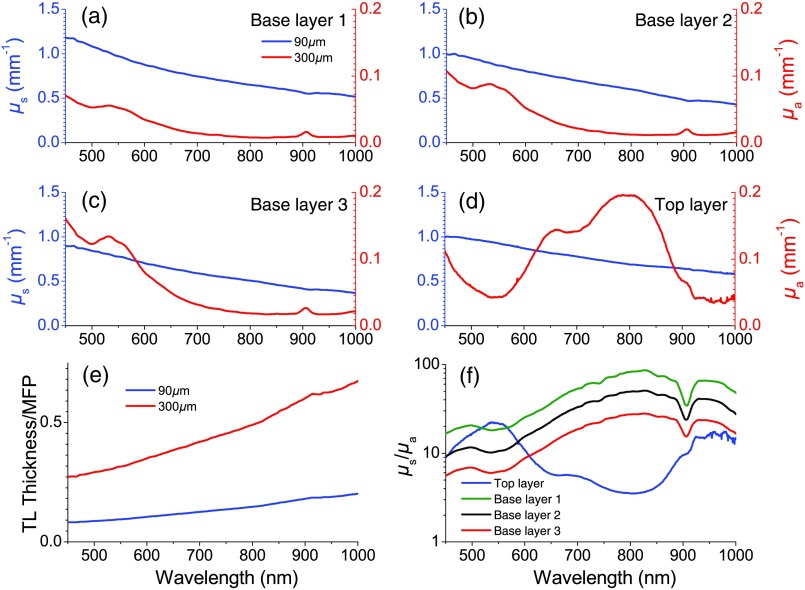
Optical properties of the phantoms. (a–d) Depict the reduced scattering coefficient (left axis) and absorption coefficient (right axis) spectra of the top layer (a) and the three possible base layers (b–d) of each phantom. (e) Provides the top layer thickness normalized by the transport mean free paths for both 90  μm (blue) and 300  μm (red) thicknesses of the top layer. (f) Depicts the relative scattering to absorption strength of each layer on a log10 scale. The green curve shows the top layer, the black shows base layer 1, the red shows base layer 2, and the blue shows base layer 3.

For this investigation, interchangeable two-layer optical phantom constructs are used to approximate the optical properties of structured tissues such as skin. These two-layer constructs provide a controlled and independently verifiable basis to evaluate the accuracy of our inverse solver. These tissue-simulating phantoms were fabricated using polydimethyl siloxane (PDMS) and follow the general procedure outlined in several previous publications.^11^^,^^25^^,^^26^ In this investigation, the bottom layer phantoms were fabricated to be 3- to 4-cm thick, which may be considered semi-infinite in terms of diffuse reflectance in the visible and near-infrared spectral regions. Specifically, freeze-dried bovine hemoglobin was used to mimic dermal absorption properties across both visible- and near-infrared ranges while titanium dioxide microparticles were used to mimic a range of tissue-relevant scattering properties. Details on the procedure for fabricating these types of hemoglobin-like phantoms are described elsewhere.^26^ Here, three bottom layer phantoms (labeled phantoms 1, 2, and 3) were fabricated using three distinct concentrations of hemoglobin. Scattering properties are also different between these phantoms but vary by only ±15% [Figs. 2(a)–2(d)].

In this study, we used phantoms of two different top layer thicknesses, 90 and 300  μm, respectively. These were fabricated from the same batch of turbid phantom material, to ensure that the optical properties of each were essentially identical. Naphthol green was selected as the absorbing agent as it provides distinct spectral features from those of the underlying hemoglobin phantoms while also providing absorption across the entire spectral range. Titanium dioxide particles were used as the scattering agent. The resulting absorption and scattering spectra for these two top layer thicknesses are shown in Fig. 2(d). Reference values for the scattering and absorption spectra of the top phantom layer were determined using integrating sphere measurements in conjunction with the inverse adding doubling method,^11^^,^^27^ whereas bottom layer optical properties were determined using SFDS measurements.^10^ Moreover, to understand these layered optical properties vis-a-vis the limitations of the SDA, in Fig. 2(e) we plot the wavelength dependence of the ratio of the reduced scattering coefficient to the absorption coefficient $\left(\mu_{s}^{\prime }/\mu_{a}\right)$ for each of these layers as well as the top layer thickness normalized to the transport mean free path in Fig. 2(f). Rather than attempt to mimic a specific tissue system, these phantoms were constructed to provide a range of optical parameters and layer thicknesses germane to layered tissue structures (e.g., epithelial tissues) and superficial tissue injury (e.g., burn wounds).^28^ Figures 2(e)–2(f) show that these properties span many ratios of scattering to absorption (from ∼4∶1 to ∼100∶1 within a given layer) and layer thickness to $l^{*}$ (from 10:7 to 10:1). Figures 2(a)–2(d) show that either the top or bottom layer can possess the dominant reduced scattering and/or absorption coefficient depending on the wavelength considered.

SFDS measurements were made on six combinations of top and bottom layer phantoms. Each of the three bottom phantoms was measured with either the 90- or 300  μm-thick phantom placed on its top surface. Prior to measurement, all air gaps between the two layers squeezed out through mechanical pressure. SFDS measurements were acquired at 51 spatial frequencies over the range of 0 to $0.5\;{\mathrm{mm}}^{-1}$ range. Three sets of measurements were acquired from each two-layer phantom configuration. Each set was carried out at a slightly displaced spatial location to average out any potential minor spatial variations in the phantom optical properties. The top and bottom layers were then separated and the top layer was then attached to the another bottom layer phantom. This procedure was performed for all combinations of top and bottom layers, resulting in data from six distinct layered phantom systems. A calibration measurement, using a reference phantom having well characterized optical properties, was performed between each layered phantom^10^^,^^24^ measurement.

The phantoms were designed with optical property ranges in absorption ($\mu_{a}=0.01$ to $0.2\;{\mathrm{mm}}^{-1}$) and reduced scattering ($\mu_{s}^{\prime }=0.5$ to $1.3\;{\mathrm{mm}}^{-1}$) coefficients that span those of many soft biological tissues in the visible and near-infrared spectral range.^28^ While the reduced scattering coefficient is dominant over absorption in both layers and across all wavelengths tested, we explore regimes where that dominance is more pronounced in either the top or the bottom layer and systems with top layer thickness far less than the transport free mean path.

### 2.3 Spatial Frequency Domain Sensitivities and Design of Multistage Optimization Algorithm

**Fig. 3:**
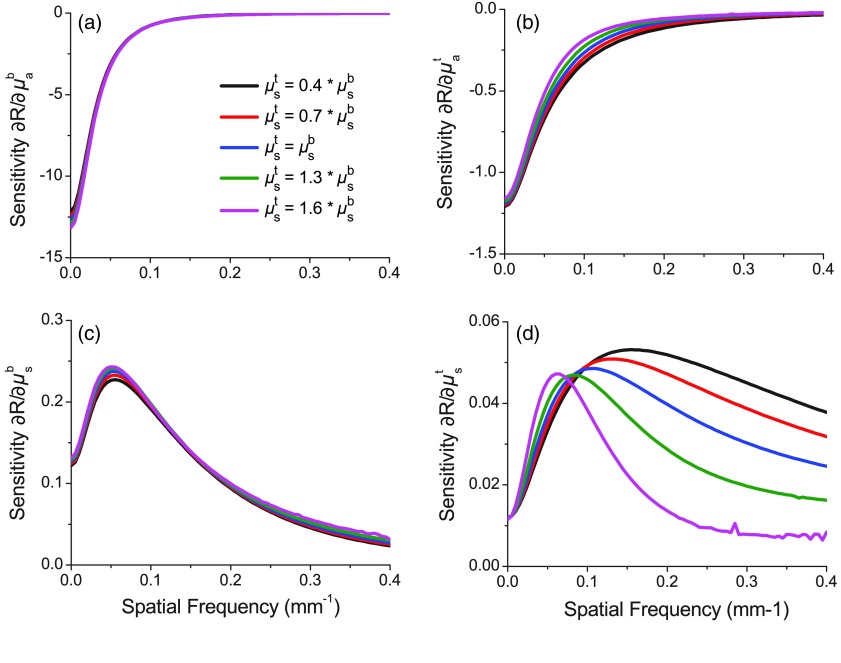
Reflectance sensitivities with respect to (a, b) absorption and (c, d) reduced scattering coefficient for (a, c) bottom and (b, d) top layers, respectively, as a function of spatial frequency for a top layer thickness of 300  μm. In each plot, the black line indicates the case where $\mu_{s,t}^{\prime }=0.4\times \mu_{s,b}$. The red line indicates $\mu_{s,t}=0.7\times \mu_{s,b}$, the blue indicates $\mu_{s,t}=\mu_{s,b}$, the green indicates $\mu_{s,t}=1.3\times \mu_{s,b}$, and the magenta indicates $\mu_{s,t}=1.6\times \mu_{s,b}$.

For analysis of this two-layer phantom system, we assume knowledge of layer thickness and attempt to recover four optical properties at each wavelength, namely the absorption and reduced scattering coefficient in each layer. *A priori* knowledge of layer thickness is a reasonable assumption as existing approaches are available for their estimation using SFDS instrumentation.^24^^,^^29^ Rather than attempting to fit all parameters simultaneously, we perform a multistage algorithm to isolate and refine the spectra of each optical property. The design of our algorithm is inspired by our prior development of multistaged inversion algorithms^8^^,^^9^ and the spatial frequency-dependent sensitivity of the measured reflectance to the absorption and reduced scattering coefficients in each layers shown below in Fig. 3.

We present the spatial frequency-dependent reflectance sensitivities with respect to each optical property for a top layer thickness of 300  μm, which is equivalent to the thicker of the layered phantom systems measured in this study. We compute these sensitivities for varying ratios of the reduced scattering coefficient between the top and bottom layers. Specifically, we fix the bottom layer properties at $\mu_{s,b}=1\;{\mathrm{mm}}^{-1}$, $\mu_{a,b}=0.1\;{\mathrm{mm}}^{-1}$ and vary top layer reduced scattering properties while matching the absorption properties with the bottom layer. The variations in top layer scattering that we explore span $\mu_{s,t}=[0.4,0.7,1,1.3,1.6]\times \mu_{s,b}^{\prime }$ with fixed top layer absorption ($\mu_{a,t}=0.01\;{\mathrm{mm}}^{-1}$).

In Figs. 3(a)–3(d), we show the spatial frequency-dependent reflectance sensitivities to (a) bottom layer absorption, (b) top layer absorption, (c) bottom layer reduced scattering, and (d) top layer reduced scattering coefficients. We note that reflectance sensitivity to either top or bottom layer absorption is increrased at the lowest spatial frequencies and decreases by an order of magnitude once spatial frequencies exceed $∼0.08\;{\mathrm{mm}}^{-1}$ for the bottom layer and $∼0.13\;{\mathrm{mm}}^{-1}$ for the top layer. Interestingly, in absolute terms, the reflectance sensitivity to top layer absorption is roughly an order of magnitude smaller than that for bottom layer absorption in this low spatial frequency regime. Moreover, the reflectance sensitivity to absorption in either layer is weakly dependent on the scattering contrast between the layers.

The spatial frequency-dependent reflectance sensitivity to the reduced scattering coefficient in either top or bottom layers shows a distinct peak at a nonzero spatial frequency. The location of the peak sensitivity for the bottom layer is fixed at $0.06\;{\mathrm{mm}}^{-1}$, whereas the location of this peak for top layer scattering shifts from 0.06 to $0.16\;{\mathrm{mm}}^{-1}$ as the scattering contrast moves from being top layer dominant ($\mu_{s,t}=1.6\times \mu_{s,b}$) to bottom layer dominant ($\mu_{s,t}=0.4\times \mu_{s,b}$). In absolute terms, the reflectance sensitivity to scattering in the bottom layer can be as much as 5× larger than the top layer sensitivity in this low spatial frequency regime. However, at higher spatial frequencies exceeding $f_{x}=0.3\;{\mathrm{mm}}^{-1}$, the reflectance sensitivity to top and bottom layer scattering becomes comparable.

These sensitivity features motivate the design of an inversion algorithm using four stages, each focused on the determination of specific optical parameters. In each stage, we consider measured reflectance values at specific spatial frequencies and 32 equispaced wavelengths spanning λ=450 to 1000 nm. Using the lsqcurvefit function in MATLAB, we seek to determine optical parameters values that minimize the least squares difference between a ${\mathrm{SHEF}}_{N}$ computation^7^ for the SFD reflectance and the actual measurements. For the ${\mathrm{SHEF}}_{N}$ computation, we consider a ninth-order expansion of spherical harmonic functions that provides a good balance between accuracy and computational expense. We will refer to this as a ${\mathrm{SHEF}}_{9}$ computation for the remainder of the paper. We also chose to consider only 32 discrete wavelengths from the multispectral dataset as it provides a good balance between spectral detail and computational expense. For the ${\mathrm{SHEF}}_{9}$ computations, we assume a single scattering anisotropy of 0.8 for both layers.^28^

The details of the four stage process are as follows:

**Stage 1: Recovery of layer-specific reduced scattering spectra.** In stage 1, we obtain estimates for the reduced scattering coefficient spectra of the top and bottom layers. Because the sensitivity to the top layer relative to the bottom layer increases at larger spatial frequencies [Figs. 3(c) and 3(d)], we choose to analyze separately SFDS data obtained at six spatial frequencies from two different spatial frequency bands ($f_{x1}=[0,0.02,\ldots ,0.1]\;{\mathrm{mm}}^{-1}$ and $f_{x2}=[0.3,0.32,\ldots ,0.4]\;{\mathrm{mm}}^{-1}$). For each frequency band, we analyze the SFDS reflectance spectra using a ${\mathrm{SHEF}}_{9}$ computation for a homogeneous medium and determine the values for absorption and reduced scattering coefficients that result in a best fit. The presumption here is that the reduced scattering properties obtained from the $f_{x1}$ spatial frequency band will be representative of the bottom layer, whereas those obtained from the $f_{x2}$ spatial frequency band will be representative of the top layer. For simplicity, we assume the spectral dependence of scattering to be governed by an inverse power law^28^$\mu_{s}=A{\left(\lambda /\lambda_{0}\right)}^{-b}$, where A is the reduced scattering coefficient for $\lambda_{0}=750\;\mathrm{nm}$. We discard predictions for optical absorption from this stage.

**Stage 2: Recovery of layer specific absorption spectra.** In stage 2, we aim to recover estimates for the absorption coefficient spectra of both top and bottom layers. We fix the reduced scattering spectra for the top and bottom layers to those obtained via the Stage 1 analysis of SFDS data in high- and low-spatial frequency bands, respectively. To obtain absorption spectra specific to both top and bottom phantom layers, we consider the SFDS spectral data at spatial frequencies of $f_{x}=0.01$ and $0.02\;{\mathrm{mm}}^{-1}$ only. We choose these spatial frequencies as our analysis in Fig. 3 reveals that the reflectance sensitivity to absorption in either layer is maximized at lower spatial frequencies. Moreover, as the reflectance sensitivity to the top layer absorption decays with spatial frequency more gradually than the bottom layer, the SFDS data at $f_{x}=0.02\;{\mathrm{mm}}^{-1}$ provide differentially more sensitivity to top layer absorption relative to that obtained at $f_{x}=0.01\;{\mathrm{mm}}^{-1}$. Given that the absorption properties of most deep tissue layers are well characterized by a linear combination of the absorption spectra of oxyhemoglobin, reduced hemoglobin, water, and lipid,^30^ we assume that the spectral shape of the absorption spectra in the bottom layer is known *a priori*. For the top layer, we adopt a more general approach and do not impose any assumptions regarding the shape of the absorption spectra. We again apply the ${\mathrm{SHEF}}_{9}$ algorithm, with a two-layer tissue geometry with known top layer thickness (90 or 300  μm). We fit the SFDS data to the ${\mathrm{SHEF}}_{9}$ predictions by determining the optimal absorption coefficient values for layers 1 and 2 while holding fixed the top and bottom layer reduced scattering coefficient spectra obtained in stage 1.

**Stage 3: Refinement of bottom layer reduced scattering spectra.** In stage 3, we aim to refine bottom layer reduced scattering spectrum obtained in stage 1. We use SFDS data obtained at $f_{x}=0.06$ and $0.15\;{\mathrm{mm}}^{-1}$. These spatial frequencies are chosen as $0.06\;{\mathrm{mm}}^{-1}$ represents the spatial frequency that is maximally sensitive to bottom layer scattering while $0.15\;{\mathrm{mm}}^{-1}$ retains significant sensitivity to bottom layer scattering while also effectively eliminating sensitivity to both top and bottom layer absorption. We fix the properties obtained from stage 1 for the reduced scattering spectrum in the top layer and from stage 2 for the top and bottom layer absorption spectra. We drop our constraint for the bottom layer reduced scattering spectra to abide by an inverse power law and perform the fit of the SFDS data to predictions provided by the layered ${\mathrm{SHEF}}_{9}$ model for each wavelength independently.

**Stage 4: Refinement of top layer absorption spectra.** The goal of the final stage is to improve the fit of the top layer absorption. As in stage 2, we again use SFDS data from $f_{x}=0.01$ and $0.02\;{\mathrm{mm}}^{-1}$ and find the top layer absorption values at each wavelength that produces predictions from our layered ${\mathrm{SHEF}}_{9}$ model that best match the data. We fix the top layer reduced scattering spectrum to that obtained in stage 1, bottom layer absorption spectrum to that obtained in stage 2, and bottom layer reduced scattering to that obtained in stage 3. For our initial guess, we use the top layer absorption spectrum obtained from stage 2.

## 3 Results

### 3.1 Stage 1: Recovery of Layer-Specific Reduced Scattering Spectra

**Fig. 4:**
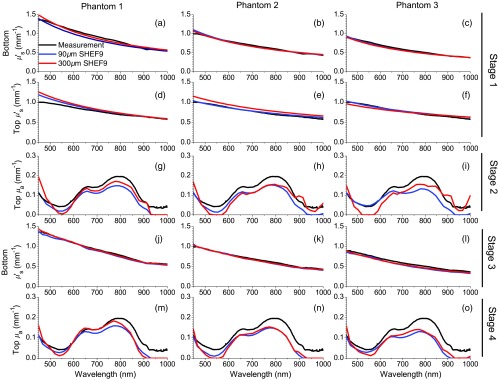
Inversion results. In all graphs, reference data are shown in black, ${\mathrm{SHEF}}_{9}$ results for a 90  μm top layer are shown in blue and ${\mathrm{SHEF}}_{9}$ results for a 300  μm top layer are shown in red. (a–f) The stage 1 results, in which initial guesses for top and bottom layer scattering spectra are recovered. (a–c) The reference scattering spectra for their respective base layers, and homogeneous ${\mathrm{SHEF}}_{9}$ results for data taken with the $f_{x1}$ spatial frequency band. (d–f) The reference scattering spectra for the top layer alone and the homogeneous SHEF results using data from the $f_{x2}$ spatial frequency band. (g–i) The top layer absorption spectra fits from stage 2, using results from stage 1 for top and bottom layer scattering. (j–l) The stage 3 results in which recovery of the bottom layer reduced scattering coefficient spectrum is refined. (m–o) The stage 4 results that provide the final result for the top layer absorption spectrum.

**Fig. 5:**
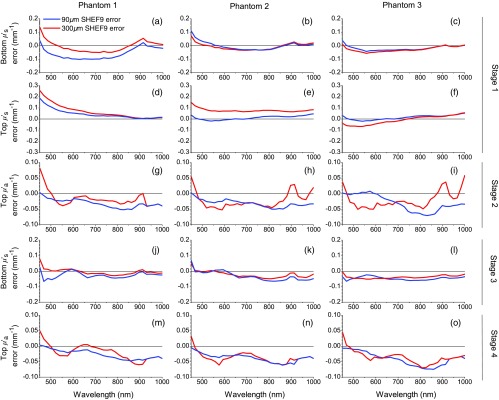
Errors of inversion results. Each subfigure shows the error for thick top layer (red) and thin top layer (blue) phantoms corresponding to the results from the same subfigure shown in Fig. 4.

**Phantom 1:** We show the results for phantom 1 in Fig. 4(a) (bottom layer scattering) and 4(d) (top layer scattering). The corresponding errors of these estimates are shown in Figs. 5(a) and 5(d), respectively. For the 300-μm-thick top layer, the error in bottom layer reduced scattering is typically $⩽0.1\;{\mathrm{mm}}^{-1}$ except for λ>450  nm where it is $=0.14\;{\mathrm{mm}}^{-1}$. The error in the recovery of the 300-μm-thick top layer reduced scattering coefficient is largest at λ=450  nm at $0.256\;{\mathrm{mm}}^{-1}$ and steadily declines for larger wavelengths. For λ>557  nm, the error in recovery of the 300-μm thick top layer reduced scattering is $<0.1\;{\mathrm{mm}}^{-1}$. For the 90  μm thin top layer phantom, error in bottom layer reduced scattering is worst at λ=628  nm, where $\mu_{s}$ is underestimated by $0.1\;{\mathrm{mm}}^{-1}$ with smaller errors occurring at all other wavelengths. The maximal error in recovery of the 90  μm thin top layer reduced scattering is $0.19\;{\mathrm{mm}}^{-1}$ at λ=450  nm and is reduced at all other wavelengths with errors $<0.1\;{\mathrm{mm}}^{-1}$ for λ⩾521  nm.

**Phantom 2:** The results for phantom 2 are shown in Fig. 4(b) (bottom layer scattering) and 4(e) (top layer scattering.) Error is shown in Fig. 5(b) for top layer scattering and in Fig. 5(e) for bottom layer scattering. For the thick top layer, the error in the bottom layer reduced scattering error is $<0.1\;{\mathrm{mm}}^{-1}$ for all wavelengths, and $<0.05\;{\mathrm{mm}}^{-1}$ for all wavelengths apart from λ=450  nm, where it is $0.076\;{\mathrm{mm}}^{-1}$. The maximal error in estimation of the 300-μm-thick top layer reduced scattering is $0.15\;{\mathrm{mm}}^{-1}$ at λ=450  nm, and falls below $0.1\;{\mathrm{mm}}^{-1}$ for λ⩾503  nm with typical errors of $0.06\;{\mathrm{mm}}^{-1}$. For the 90-μm-thin top layer, a maximal error in the estimation of the bottom layer reduced scattering coefficient is $0.11\;{\mathrm{mm}}^{-1}$ occurring once again at λ=450  nm. For λ⩾503  nm, this error is $<0.05\;{\mathrm{mm}}^{-1}$. Top layer reduced scattering error for the 90-μm-thin top layer is within $\pm 0.05\;{\mathrm{mm}}^{-1}$ at all wavelengths.

**Phantom 3:** Figures 4(c) (bottom layer scattering) and 4(f) (top layer scattering) provide the results from phantom 3. Error is shown in Figs. 5(c) and 5(f), respectively. For the 300-μm-thick top layer, error in the recovery of the bottom layer reduced scattering is between $-0.05\;{\mathrm{mm}}^{-1}$ and 0 at all wavelengths. Top layer reduced scattering error remains within $\pm 0.07\;{\mathrm{mm}}^{-1}$. For the 90-μm-thin top layer, phantom for bottom layer reduced scattering is underestimated by $⩽0.05\;{\mathrm{mm}}^{-1}$ for all wavelengths. Error in the top layer reduced scattering is within $\pm 0.05\;{\mathrm{mm}}^{-1}$ for all wavelengths.

### 3.2 Stage 2: Recovery of Layer-Specific Absorption Spectra

In stage 2, we determine the magnitude of a predefined spectral shape for bottom layer absorption and an initial estimate for the top layer absorption spectrum. Table 1 shows the results for bottom layer absorption coefficient for each phantom. A recovered coefficient value of β=1 represents perfect recovery. For all phantoms with the 300-μm-thick top layer, error in the recovery of $\mu_{a}$ was <5%. For phantoms 1 and 2, the error is <1%, and for phantom 3, the error is ∼4.5%. For the 90-μm-thin layer phantoms, we recover the β value with <9% error overall. Specifically, the error in the estimation of the bottom layer absorption in phantoms 1, 2, and 3 being 3.6, 6.2, and 8.1%, respectively.

**Table 1:** Coefficients (β) of the assumed absorption spectrum recovered for each base layer choice and top layer thickness. As the spectrum being fit is the reference data for bottom layer absorption, β=1 indicates perfect recovery.

Base layer	90-μm top layer β	300-μm top layer β
1	1.04	1.00
2	0.94	0.99
3	0.92	0.95

We show the initial estimates for the top layer absorption in Figs. 4(g)–4(i). It is important to remember that these estimates are provisional; the top layer absorption spectra obtained here will be refined in stage 4. We comment on the accuracy of these estimates at four sets of wavelengths: λ=450  nm, 450 to 600 nm, 600 to 850 nm, and >850  nm. For λ=450  nm, recovery of $\mu_{a}$ for the 300-μm-thick top layer phantom is overestimed between 0.04 and $0.08\;{\mathrm{mm}}^{-1}$, whereas the 90-μm-thin top layer phantoms experience very small errors ($⩽0.01\;{\mathrm{mm}}^{-1}$). For λ=450 to 600 nm, the reference data for absorption drops to $\mu_{a}⩽0.07\;{\mathrm{mm}}^{-1}$, at which point the method experiences difficulties in recovering the correct value, which often results in an extreme underestimation of the absorption. Typically, recovery of $\mu_{a}$ for the 300-μm-thick top layer is worse than for the 90-μm-thin layer. Here, error for $\mu_{a}$ in 300-μm-thick top layer phantoms is between 0.04 and $0.05\;{\mathrm{mm}}^{-1}$, whereas that of the 90  μm-thin top layer phantoms is near $0.03\;{\mathrm{mm}}^{-1}$ for phantoms 2 and 3, respectively. Interestingly, recovery of $\mu_{a}$ for the 300-μm-thick top layer in Phantom 1 is accomplished with much smaller error than its counterparts with phantoms 2 and 3, whereas the recovery of $\mu_{a}$ in phantom 3 with the 90-μm thin layer has negligible error. For λ=600 to 850 nm, the reference data for $\mu_{a}$ experience a double hump with values between 0.01 and $0.02\;{\mathrm{mm}}^{-1}$. Despite some underestimation, this spectral structure is well recovered in the inversion results for each phantom. Errors for $\mu_{a}$ in 300-μm thick top layer phantoms in this region are between 0.02 and $0.05\;{\mathrm{mm}}^{-1}$, with best performance for phantom 1. Cases with the 90  μm-thin layer have larger error for $\mu_{a}$ at the shorter wavelengths and less at longer wavelengths when compared with 300-μm-thick top layer phantoms. These error rates stay in the same general range as those for the 300-μm-thick top layer phantoms but tend more toward $0.05\;{\mathrm{mm}}^{-1}$. For λ>850  nm, the reference data for $\mu_{a}$ experience a drop off similar to when λ=450 to 600 nm, falling as λ increases until it levels off near $0.05\;{\mathrm{mm}}^{-1}$. We see a similar underestimation for $\mu_{a}$ in most phantoms in this spectral regime, though here it is more pronounced in the cases with the 90-μm-thin top layer. Interestingly, when phantoms 2 and 3 have 300-μm thick top layers, the inversion results are less stable, involving a peak overestimation for $\mu_{a}$ near λ=900  nm and a peak underestimation near λ=950  nm. Error for $\mu_{a}$ here ranges for all phantoms between $\pm 0.05\;{\mathrm{mm}}^{-1}$. When considering the combined results across all wavelengths, it is important to note that the general shape of the $\mu_{a}$ spectrum is recovered in each phantom, generally within $\pm 0.05\;{\mathrm{mm}}^{-1}$, and significant underestimations are made when $\mu_{a}<0.075\;{\mathrm{mm}}^{-1}$. Interestingly, recovery of the absoption spectra for the 90-μm-thin top layer phantoms tends to experience smaller absolute errors than for the 300-μm-thick top layer phantoms when λ⩽700  nm, whereas the opposite is true for λ>700  nm.

### 3.3 Stage 3: Refinement of Bottom Layer Reduced Scattering Spectra

The aim of stage 3 is to improve the initial estimates for bottom layer reduced scattering coefficient spectra obtained in stage 1. We show the results for stage 3 in Figs. 4(j)–4(l), and the relative error rates in Figs. 5(j)–5(l). In all phantoms, error in the recovery bottom layer $\mu_{s}^{\prime }$ was reduced at nearly all wavelengths and with structure of the scattering spectra more faithfully recovered as compared with the results in stage 1. Figure 4(j) shows the results for phantom 1. The reduced scattering coefficient spectrum for the 300-μm-thick top layer phantom was recovered with an absolute error of $⩽0.02\;{\mathrm{mm}}^{-1}$ with the exception of an error of $0.06\;{\mathrm{mm}}^{-1}$ at λ=450  nm. The 90-μm-thin top layer phantom performed slightly worse, with an underestimation of the reduced scattering coefficient by $0.07\;{\mathrm{mm}}^{-1}$ at λ=450  nm and errors of $\pm 0.04\;{\mathrm{mm}}^{-1}$ at all other wavelengths. Figure 4(k) shows the results for phantom 2. The absolute error for the reduced scattering coefficient for thick top layer phantom was limited to $\pm 0.05\;{\mathrm{mm}}^{-1}$ except for λ=450  nm where the error was $0.06\;{\mathrm{mm}}^{-1}$. Recovery of the reduced scattering coefficient for thin top layer phantom was slightly better with error of $\pm 0.05\;{\mathrm{mm}}^{-1}$. Figure 4(l) shows results in the recovery of the bottom layer reduced scattering for phantoms 3. The performance for both thick and thin top layers is similar with scattering estimated with an error of $⩽0.05\;{\mathrm{mm}}^{-1}$ across the full spectral region.

### 3.4 Stage 4: Refinement of Top Layer Absorption Spectra

The aim of stage 4 is to improve the estimates for top layer absorption obtained in stage 2. We show these results Figs. 4(m)–4(o) and the absolute error of these estimates in Figs. 5(m)–5(o). We consider these results relative to those obtained in $\mu_{a}$ in stage 2.

**Phantom 1:** The recovered absorption spectra for the top layers in phantom 1 are shown in Fig. 4(m), with error in Fig. 5(m). We obtain improvements in the predicted absorption spectra for both thick and thin layers as compared with the results obtained in stage 2 [shown in Figs. 4(g) and 5(g)]. The overestimation for $\mu_{a}$ found in the case of the thick top layer at λ=450  nm is $∼0.03\;{\mathrm{mm}}^{-1}$, whereas the error remains minimal for the case of the thin top layer. We see that error for $\mu_{a}$ is likewise reduced for both the thin and thick top layer cases with improvements between 0 and $0.02\;{\mathrm{mm}}^{-1}$ in the λ=450 to 600 nm spectral region. We see greater improvements in $\mu_{a}$ error in the λ=600 to 850 nm wavelength intervals, particularly for the thick top layer with improved accuracy with $\mu_{a}$ errors generally below $\pm 0.03\;{\mathrm{mm}}^{-1}$ for the thick top layer and $0.04\;{\mathrm{mm}}^{-1}$ for the thin top layer. For λ>850  nm, we obtain slightly worse results until λ=920  nm after which the errors obtained in stages 2 and 4 are essentially equivalent. The absorption spectra obtained for phantom 1 in stage 4 improve upon stage 2 for the entire interval of λ=450 to 920 nm, beyond which the recovery is equivalent. Errors in the recovered top layer absorption are limited to $⩽0.04\;{\mathrm{mm}}^{-1}$ for λ=450 to 920 nm.

**Phantom 2:** Recovery of top layer absorption spectra in phantom 2 is shown in Fig. 4(n), with absolute error shown in Fig. 5(n). For the case with the thick top layer, we see overall improvement in capturing the shape of the absorption spectra with slight reductions in the absolute error. This is seen particularly as a lower (by $0.02\;{\mathrm{mm}}^{-1}$) overestimation of $\mu_{a}$ when λ=450  nm and a less pronounced underestimation in the λ=450 to 600 nm range (despite having a similar minimum). The double hump of $\mu_{a}$ is once again captured in the λ=600 to 850 nm spectral range, with smaller (between 0 and $0.01\;{\mathrm{mm}}^{-1}$ reduction) underestimation for the thick top layer phantom. For λ>850  nm, both thick and thin top layer phantoms experience a sharp decline as λ increases, ending in the near 0 values observed earlier. This is in contrast to the thick top layer performance from stage 2, which involved an overestimation of $\mu_{a}$ when λ=880 to 920 nm. Errors remain similar in absolute value to those of stage 2 but are more uniform across wavelength and better represents the overall shape of the reference absorption spectrum.

**Phantom 3:** Recovery of top layer absorption spectra in phantom 3 is shown in Fig. 4(o), with absolute error shown in Fig. 5(o). For the thick top layer phantom, the initial overestimation at λ=450  nm is actually worse, increasing to $0.05\;{\mathrm{mm}}^{-1}$ as compared with $0.04\;{\mathrm{mm}}^{-1}$ in stage 2. While the absolute error is worse in many spectral regions, the recovered spectral shape is better captured as compared with stage 2, and we underestimate the absorption by only $0.05\;{\mathrm{mm}}^{-1}$ in the case of the thick top layer phantom at λ=588  nm, with a smaller underestimation ($0.03\;{\mathrm{mm}}^{-1}$) for the thin top layer phantom. Absolute error in absorption stabilizes to within $\pm 0.04\;{\mathrm{mm}}^{-1}$ for λ=605 to 795 nm. For longer wavelengths, we recover a more accurate spectral shape for the case of the thick top layer and comparable results for the case of the thin layer as compared with stage 2. This manifests in the same way it does for phantom 2, with an elimination of the overestimation found in stage 2, once again resulting in a more accurate recovery of the spectral structure of $\mu_{a}$.

## 4 Discussion

Our method demonstrates the broad recovery of the bottom layer absorption and reduced scattering coefficients as well as the top layer reduced scattering coefficients across all wavelengths. Moreover, there are specific spectral regions in which the top layer absorption is reliably recovered. Specifically, bottom layer values for the reduced scattering coefficient are generally within 5% of the reference values. Moreover, bottom layer absorption coefficients are typically recovered within the 5% range with a worst-case error of 8.1%. Similarly, the top layer reduced scattering coefficient values obtained in stage 1 are typically within 10% of known values. In each case, the general spectral shape of the top layer reduced scattering coefficient is recovered. Typically, the maximum error for the top layer reduced scattering coefficient is observed at λ=450  nm, which may be indicative of the limitations of using a single inverse power law across the entire wavelength range. Attempts to further improve the fit for top layer scattering without *a priori* assumption for the spectral shape of the top layer reduced scattering coefficient have not been reliably successful. We believe that these problems stem primarily from the intrinsically low measurement sensitivity to top layer parameters as shown in Fig. 3. Specifically, the reflectance sensitivities to top layer reduced scattering and absorption coefficients are roughly an order of magnitude smaller than the corresponding bottom layer parameters and compromises our ability to obtain accurate measures for top layer optical properties. However, we note that the spatial frequency at which we have maximal sensitivity to the top layer reduced scattering coefficient [Fig. 3(d)] changes based on the scattering contrast between the two layers. Therefore, improvements in the recovery of top layer scattering in stage 3 may be obtained by an inversion approach that adaptively selects data from different spatial frequencies. These frequency selections must provide sufficient sensitivity and specificity to enable differentiation between top and bottom layer properties based upon preliminary estimates obtained from stage 1.

The greatest need for improvement of our algorithm pertains to the recovery of the top layer absorption spectra. It should be noted that the top layer thicknesses tested have exceeding small absorption optical densities in the range $10^{-3}$ to $10^{-1}$. We believe that the difficulties in the recovery of top layer absorption stem from the same causes that hamper the recovery top layer reduced scattering. The recovery of the top layer absorption is further compromised by the fact that the reflectance sensitivity to both top and bottom layer absorption is maximized at the similar spatial frequencies. This limits the use of spatial frequency selection to provide differential absorption sensitivity to one layer versus the other. Unfortunately, the shapes of absorption spectra vary greatly between different molecules as compared with scattering spectra for different scatterers.^31^ Moreover, the greater multiplicity of absorbers that reside in superficial tissue layers^32^ preclude the use of simplifying assumptions for the spectral shape of the top layer absorption coefficient. The most problematic feature in the recovered absorption coefficient spectra is the near-zero value that appears at many wavelengths.

Interestingly, the recovery of near zero absorption values occurring for λ=520 to 570 nm for 300-μm layer phantoms does not appear as commonly in the results for the 90-μm layer phantoms. This is counterintuitive, though it may be due to the fact that a larger absorption coefficient in thin layer will have a similar impact on the measured reflectance as a smaller absorption coefficient in a thicker layer. This, combined with smaller errors in other parameters, may explain why we do not see such severe underestimates in the top layer absorption for the 90-μm layer phantoms at these wavelengths. While we can find no single perfect predictor for this phenomenon in any reference spectrum, it appears only when the top layer absorption falls below $0.075\;{\mathrm{mm}}^{-1}$. When top layer absorption is above these values, results are much better, with typical errors in the 15% to 30% range. Indeed, the refinement in the top layer absorption spectra in stage 4 generally results in improved recovery, particularly in the λ=450 to 600 nm spectral region. For λ=600 to 850 nm, we generally observe reduction in error for all phantoms. This wavelength interval carries useful information for chromophores such as oxy and deoxyhemoglobin, and our method produces reliable results in this wavelength range. It is also possible that the use of oblique incident radiation and measurement of spatial phase shifts may provide more sensitivity to top layer optical parameters,^7^^,^^33^ and this may be a subject for future investigation.

Comparison of our results to other studies using analytical approaches is challenging as none have provided accurate results in systems where the top layer thickness $≲l^{*}$ while we have exclusively focused on layered systems with top layer thicknesses in the range of 0.1 to $0.8l^{*}$. The performance of our staged algorithm competes favorably as compared with those reported in previous perturbation Monte Carlo methods.^18^ However, those studies focused on optical property recovery at a single wavelength as opposed to multispectral recovery of optical properties. Published studies using LUT-based methods^21^^,^^23^ involved biologically simplifying assumptions such as uniform scattering properties across all layers. Such methods will also have difficulty as the number of unknown parameters increases, due to the relationship between dimensionality of the required lookup table and the requirements to generate and store such tables. Multistage algorithmic approaches using nonlinear optimization methods such as the one that we have presented should experience more linear growth in complexity as additional parameters are introduced. In such algorithms, each stage provides an initial estimate for the parameter values or refines an earlier fit for a limited number of parameters, rather than having to determine all the unknown parameters simultaneously. Moreover, our ${\mathrm{SHEF}}_{9}$ forward solver can easily implement alternate single-scattering phase functions without changes in the underlying inversion algorithm. By contrast, consideration of alternate single-scattering phase functions or even a different single scattering anisotropy value would necessitate perturbation MC or LUT methods to generate entirely new sets of photon biographies or tables.

## 5 Conclusion

We present a staged inversion algorithm using an approximate deterministic RTE solver to recover the optical properties of layered media using SFDS data over a broad range of wavelengths. Our approach assumes a known top layer thickness and knowledge of the wavelength dependence of the bottom layer absorption coefficient. Bottom layer absorption and reduced scattering coefficients are recovered within 10% error, with the majority of error being <5%. Top layer absorption properties are recovered with 15% to 30% error within the λ=600 to 800 nm spectral window. This method provides accuracy comparable with current perturbation Monte Carlo methods that have provided recovery of optical properties at single wavelengths and is able to recover the optical properties of a top layer with thickness down to $l^{*}/10$.

This method is scalable to allow the recovery of optical properties at any number of wavelengths. In this regard, the ability to perform fits on multi-/hyperspectral datasets is limited solely by the available computational power and need for rapid processing. If computational power is limited, future work to improve the efficiency of stage 2 should be prioritized as this stage involves a multispectral inversion of dimension equal to the sum of the number of wavelengths in the dataset and the number of chromophore concentrations to be determined. This is in contrast to the lower dimensional fits performed in all other stages of this inversion scheme.

Our method achieved these levels of accuracies while requiring far less computational expense when compared with current Monte Carlo based methods. Moreover, our approach dispenses with many unrealistic assumptions used in the construction of Monte Carlo lookup tables, for example, assuming uniform scattering properties across layers. The use of the ${\mathrm{SHEF}}_{N}$ radiative transport solver^7^ provides a computational flexibility that allows for the use of different scattering phase functions with ease. In addition, the lack of a lookup table and more modest growth in computational expense with an increase in the number of unknown parameters potentially allows this approach to be applied to tissues of much greater complexity. When compared with other analytic solvers, particularly those using the SDA, we have enabled the capacity to obtain optical properties from both layers of a multilayered tissue with layer thicknesses $<l^{*}$.^13^^,^^14^^,^^34^ In fact, we are unaware of other existing approaches that make use of analytic solvers to recover optical absorption and scattering parameters layered tissues with characteristic layer thickness $<l^{*}$ from multispectral data.

Future work to improve accurate recovery of the top layer absorption spectrum will focus on eliminating the need to assume a known top layer thickness, which is common across many methods.^18^^,^^19^^,^^21^ The elimination of these assumptions would be useful for any application that relies on sensitive and accurate recovery of these parameters. We believe that algorithms that implement adaptive selection of data at specific spatial frequencies based on reflectance sensitivity characteristics would be of particular use, particularly as these characteristics vary significantly with the ratio of reduced scattering between the top and bottom layers. The selection of optimal spatial frequencies when using an adaptive inversion approach can be informed by the calculation of sensitivity curves such as those shown in Fig. 3.
